# Interactive Organization of the Circadian Core Regulators PER2, BMAL1, CLOCK and PML

**DOI:** 10.1038/srep29174

**Published:** 2016-07-07

**Authors:** Takao Miki, Zhaoyang Zhao, Cheng Chi Lee

**Affiliations:** 1 Department of Biochemistry and Molecular Biology, Medical School, University of Texas Health Science Center-Houston, Houston, Texas 77030, USA.

## Abstract

The BMAL1 and CLOCK heterodimer in the mammalian circadian transcriptional complex is thought to be repressed by PER2 and CRY1 via direct interactions. We recently reported that PER2 is largely cytosolic in *Pml*^*−*/*−*^ cells and did not co-immunoprecipitate (co-IP) with BMAL1 or CLOCK. Here, using multi-color immunofluorescence (IF) staining and co-IP, we observed a nuclear distribution of BMAL1 and a predominately cytosolic distribution of CLOCK in *Pml*^*−*/*−*^ MEF. In the presence of WT PML, PER2 co-localized with BMAL1 in the nucleus. In *Pml*^*−*/*−*^ MEF transfected with mutant K487R PML, we observed that BMAL1 and PER2 co-localized with K487R PML in the cytosol. Furthermore, cytosolic CLOCK and PER2 displayed a significant non-overlapping IF staining pattern. In *Bmal1*^*−*/*−*^ MEF, CLOCK was primarily cytosolic while PML and PER2 were nuclear. Together, our studies suggest that PML mediates the binding of PER2 to BMAL1 in the BMAL1/CLOCK heterodimer and is an important component in the organization of a functional clock complex in the nucleus. Our studies also support that BMAL1 is important for CLOCK nuclear localization.

The evolutionarily conserved circadian clock is a fundamental mechanism in most life forms, a biological response to Earth’s diurnal cycles. The transcription/translation feedback loops of the mammalian circadian clock sustain the oscillating expression pattern of the key clock genes and clock output genes. In the core loop, the BMAL1/CLOCK heterodimer binds to the E-boxes in the promoters of Per and Cry genes and drives their expression. The prevailing view is that the protein products of the Per and Cry genes form a PER/CRY dimer that enters the nucleus and represses BMAL1/CLOCK-mediated expressions of clock target genes^1^. The circadian regulator PERIOD 2 (PER2) was identified almost 2 decades ago^2^,^3^. We have reported that *Per2* mutant and null mice have disrupted circadian rhythm behaviors^2^,^4^. At the molecular level, PER2 deficient mice displayed a reduced expression of core clock genes in the suprachiasmatic nucleus (SCN) and in peripheral tissues suggesting that PER2 acts as a positive regulator of the clock transcriptional mechanism *in vivo*^5^. Indeed, several independent studies have now supported PER2’s role as a positive regulator of BMAL1/CLOCK mediated transcription. A recent study demonstrated that PER2 acts as a positive regulator via PER2 inhibition of CRY1 mediated repression of BMAL1/CLOCK transcriptional activity^6^. These observations are consistent with biochemical reconstitution studies using purified recombinant proteins that demonstrated that PER2, in contrast to CRY1 and CRY2, apparently does not interact with the BMAL1/CLOCK:DNA complex^7^. Rather the study reported that PER2 serves to remove CRY1 from binding the BMAL1/CLOCK:DNA complex. These observations raised the possibility that PER2’s interaction with BMAL1 or CLOCK is not direct but rather through intermediary partner(s). Consistent with the notion that PER2 requires an intermediary partner to interact with the BMAL1/CLOCK transcriptional complex, we have previously reported that in the absence of promyelocytic leukemia (PML) protein, immunoprecipitation (IP) of PER2 did not bring down BMAL1 or CLOCK^8^. The PML protein has been implicated in many important biological processes such as the DNA damage response, cell division control, chromosome instability and circadian rhythm^8^,^9^. In addition, *Pml* gene translocation with RARα is the major underlying cause of acute promyelocytic leukemia (APL)^10^. Our studies revealed that PML is an important circadian clock regulator^8^. We observed that *Pml*^*−*/*−*^ mice have an abnormal phase shift response to a light pulse and have a period length that displays reduced precision and stability. At the molecular level, the loss of PML disrupts PER2 nuclear localization and dampens BMAL1/CLOCK mediated transcription. That the absence of PML leads to alteration of PER2 nuclear localization presented an opportunity to determine whether PER2 interaction with the BMAL1/CLOCK heterodimer complex is direct or occurs via an intermediary partner(s).

PML is known to be able to form PML nuclear bodies (PML-NB)^11^. It has long been suggested that PML-NB’s are macromolecular scaffolds that modulate post-translational modifications and availability of many nuclear proteins with various cellular functions^12^. In our earlier study, we observed that PML enhances BMAL1/CLOCK transcriptional activity only in the presence of PER2^8^, suggesting that PML may be involved in PER2/BMAL1/CLOCK complex formation. Here we investigate how PML organizes PER2/BMAL1/CLOCK complex formation. We undertook immunofluorescence (IF) and co-immunoprecipitation (co-IP) studies using *Pml*^+/+^ and *Pml*^*−*/*−*^ cells to investigate the cellular distribution and protein-protein interactions of PER2 with BMAL1 and CLOCK. Our studies revealed that PML mediates the binding of PER2 to BMAL1 in the BMAL1/CLOCK heterodimer. Our studies also revealed that BMAL1 but not CLOCK is pivotal for BMAL1/CLOCK heterodimer interaction with PML. We observed that the cellular distribution of PER2 and CLOCK but not BMAL1 is influenced by PML. Using *Bmal1*^*−*/*−*^ MEF, we observed that nuclear distribution of CLOCK but not PML is influenced by BMAL1.

## Results

### PML mediates BMAL1-PER2 interaction

**Figure 1:**
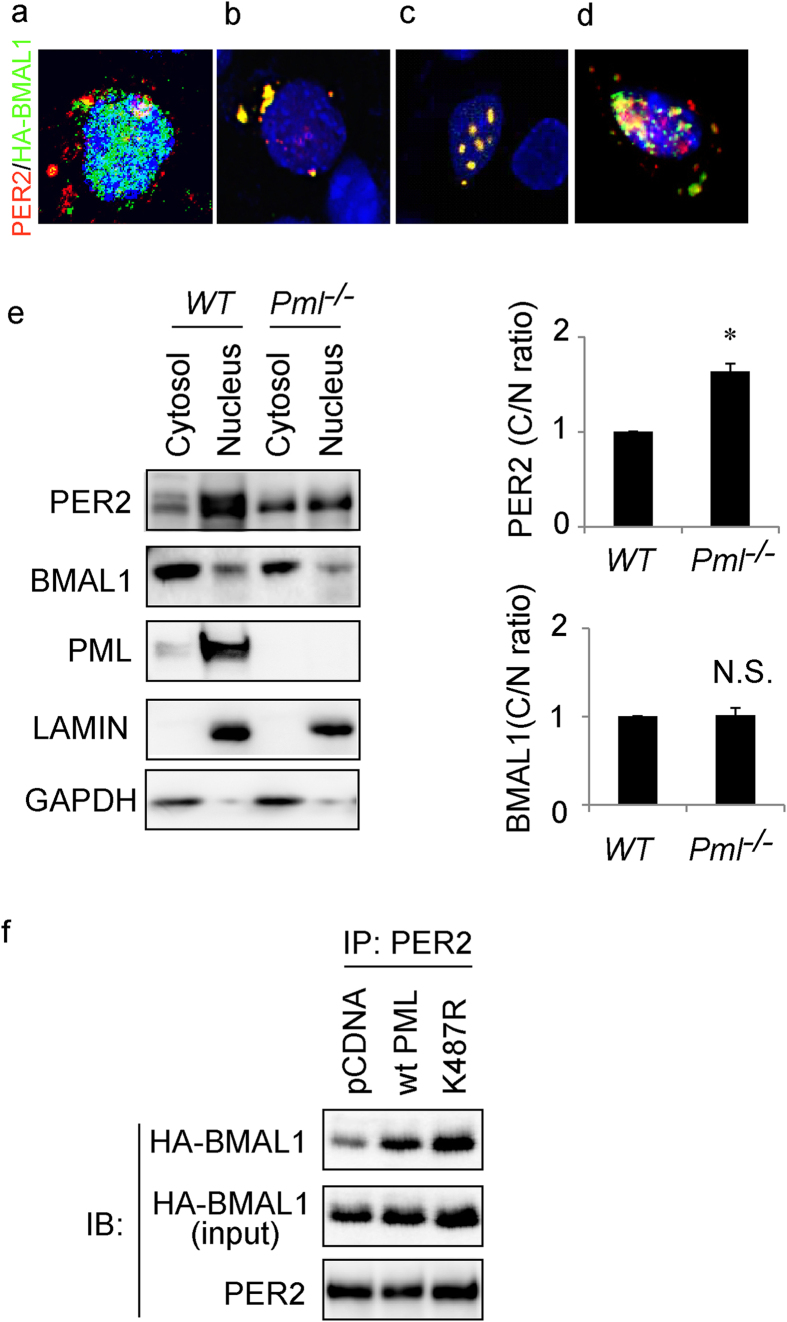
Immunofluorescence and immunoprecipitation analyses of PML’s influence on PER2 and BMAL1 interaction. Immunofluorescence analysis and DAPI staining of *Pml*^*−*/*−*^ MEF transfected with plasmids expressing (**a**) PER2 (red) and HA-BMAL1 (green). (**b**) K487R PML, PER2 (red) and HA-BMAL1 (green). (**c**) PML, PER2 (red) and HA-BMAL1 (green). (**d**) WT MEF cells transfected with plasmids expressing PER2 (red) and HA-BMAL1 (green). We observed more than 20 cells and pictures are representative images. (**e**) Left: western analysis of cytosol and nuclear fractions for wild type and *Pml*^*−*/*−*^ MEF transfected with plasmid expressing PER2 and BMAL1 using the respective antibodies for PER2 and BMAL1. Right: statistical analysis of cytosol (C)/nuclear fractions (N) ratio of indicated proteins. WT MEF cells were set to 1.0. Bars: SEM. P < 0.01. N.S.: not significant. n > 3. (**f**) Western analysis of IP of *Pml*^*−*/*−*^ MEF transfected with the respective plasmids *pcDNA, Wt Pml* and *K487R Pml* using the appropriate antibodies for PER2 and HA (n = 2).

In our previous study we reported that in the absence of PML, PER2 did not co-IP with either BMAL1 or CLOCK^8^. To expand on this finding, we undertook immunofluorescence (IF) studies to investigate whether the loss of PML affects the cellular distribution of BMAL1 in cells. Expression constructs for HA-tagged *Bmal1* and *Per2* were transfected into *Pml*^*−*/*−*^ MEF cells. IF staining was carried out with antibodies against HA epitopes and PER2. Secondary antibodies with different colored fluorescent dye tags were used to visualize the subcellular distribution of the expressed proteins. In the absence of PML, we observed that BMAL1 maintained a strong nuclear distribution with only a limited amount in the cytosol (Fig. 1a). By contrast, PER2 (red) was localized primarily in the cytosol as previously observed^8^. To ascertain whether the expressed protein was in the nucleus or in the cytosol, the BMAL1 cellular distribution was further analyzed by western blotting following cell fractionation. Western analysis with anti-BMAL1 antibodies showed that the cellular distribution of BMAL1 was not altered by the loss of PML. BMAL1 protein was detected in both nuclear and cytosolic fractions and their relative cellular distribution was unchanged in the presence or absence of PML (Fig. 1e). In contrast, PER2 cellular distribution was primarily nuclear in the presence of PML, but become highly cytosolic in the absence of PML (Fig. 1a,c,d,e).

Previously, we have reported that mutant K487R PML does not enter the nucleus and appears bound to the cytosolic side of the nuclear membrane^8^. Therefore, we examined whether introducing K487R PML affects the HA-BMAL1 distribution pattern in *Pml*^*−/−*^ MEF. Expression constructs for *HA-Bmal1, Per2* and *K487R-Pml* were transfected into *Pml*^*−*/*−*^ MEF. Immunostaining with the respective anti bodies revealed that PER2 and BMAL1 were now both co-localized outside the nucleus when K487R PML was expressed in *Pml*^*−*/*−*^ MEF (Fig. 1b). To confirm that PML, BMAL1 and PER2 were interacting, we undertook co-IP studies with anti-PER2 antibodies using cell extracts prepared from these transfections. We observed that the anti-PER2 antibody co-IPed both PER2 and BMAL1 in the presence of either PML or K487R PML (Fig. 1f). These studies revealed that PER2 and BMAL1 formed a complex with PML regardless of whether PML was located in the nucleus or in the cytosol. Together, these studies demonstrate that BMAL1 interaction with PER2 is enhanced by PML.

### CLOCK does not directly interact with PER2

**Figure 2:**
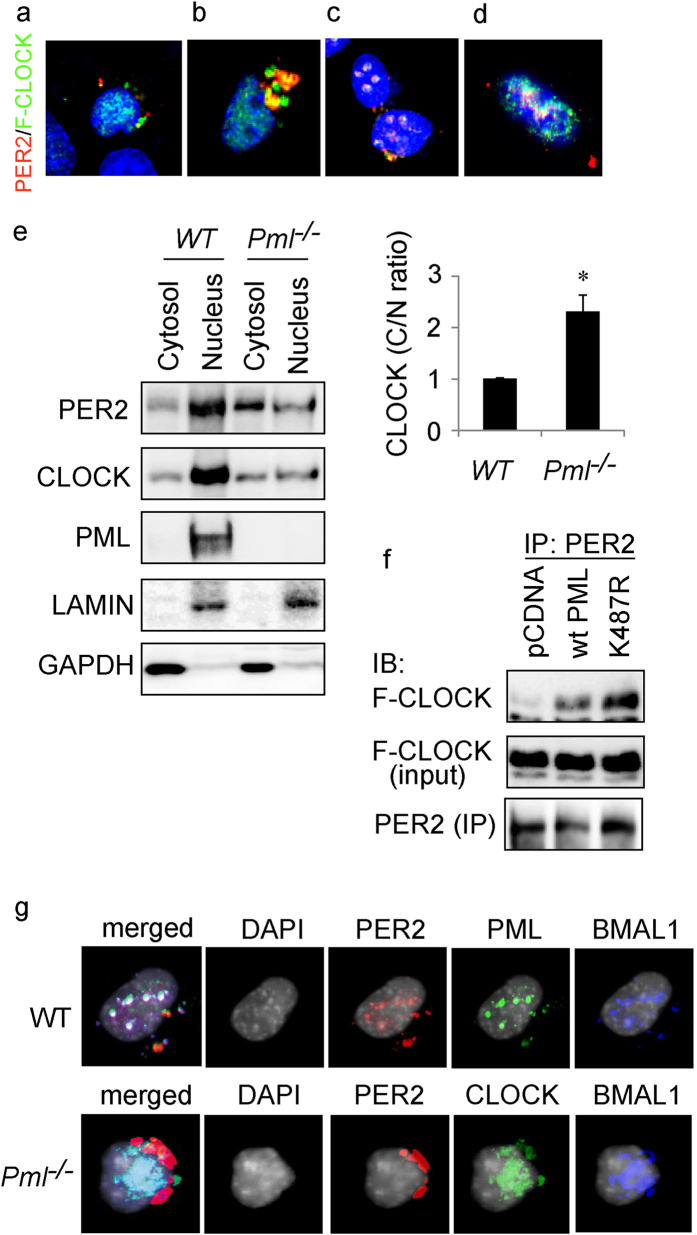
Immunofluorescence and immunoprecipitation analysis of PML’s influence on PER2 and CLOCK interaction. Immunofluorescence analysis of *Pml*^*−*/*−*^ MEF transfected with plasmids expressing (**a**) PER2 (red) and FLAG-CLOCK (green). (**b**) K487R PML, PER2 (red) and FLAG-CLOCK (green). (**c**) PML, PER2 (red) and FLAG-CLOCK (green). (**d**) WT MEF cells were transfected with plasmids expressing PER2 (red) and FLAG-CLOCK (green). (**e**) Left: western analysis of cytosol and nuclear fractions for wild type and *Pml*^*−*/*−*^ MEF using the respective antibodies for PER2, CLOCK and PML. Right: statistical analysis of the cytosol (C)/nuclear fraction (N) ratio. WT MEF cells were set to 1.0. Bars: SEM. P < 0.05. n = 4. (**f**) Western analysis of IP of *Pml*^*−*/*−*^ MEF transfected with the respective plasmids *pcDNA, Wt Pml* and *K487R Pml* using the appropriate antibodies for PER2 and FLAG. (n = 2) (**g**) Immunofluorescence analysis with the indicated antibodies of WT MEF transfected with plasmids expressing (upper panel) *Wt Pml, Per2* and *Bmal1*. Lower panel: *Pml*^*−*/*−*^ MEF transfected with plasmids expressing *Per2, Flag-Clock* and *Bmal1*. We observed more than 20 cells and pictures are representative images.

Next, we investigated whether the cellular distribution of CLOCK is altered by the loss of PML. Expression constructs for *Flag-Clock* and *Per2* were transfected into *Pml*^*−*/*−*^ MEF. Immunofluorescence staining with anti-Flag antibodies revealed that CLOCK was observed in both the nucleus and cytosol of PML deficient cells (Fig. 2a). The distribution of CLOCK in the cytosol was moderately enhanced in *Pml*^*−*/*−*^ cells compared to wild type (Fig. 2a,c,d). To verify the CLOCK cellular distribution determined by IF, we fractionated the transfected *Pml*^*−*/*−*^ and WT cells into nuclear and cytosolic fractions and undertook western analysis with anti-CLOCK antibodies. Consistent with the immunofluorescence findings, western analysis revealed a greater level of CLOCK was present in the cytosol than in the nuclear fractions in *Pml*^*−*/*−*^ cells, while CLOCK was predominantly nuclear in WT cells (Fig. 2e). IF studies with both anti-PER2 and anti-FLAG antibodies revealed that PER2 and CLOCK were co-localized in the nucleus when wild type PML was additionally co-transfected into *Pml*^*−*/*−*^ MEF (Fig. 2c). Consistent with the immunofluorescence observations, western analysis of cell fractions with anti-FLAG antibodies revealed that the majority of CLOCK protein was present in the nuclear fraction when wild type PML was present (Fig. 2e). The PER2 IF signals were largely observed in the cytosol of PML deficient MEF as previously observed, but became nuclear in the presence of PML (Fig. 2a,c,d). Interestingly, in *Pml*^*−*/*−*^ MEF a significant amount of the IF staining patterns of cytosolic PER2 (red) and CLOCK (green) appear independent, suggesting that cytosolic PER2 and CLOCK may not directly interact. These observations are consistent with our previous findings that CLOCK does not co-IP with PER2 in the absence of PML^8^. These findings further suggest that cytosolic PER2 and CLOCK interaction requires PML. To address this possibility, we transfected expression constructs for *K487R Pml, Clock* and *Per2* into *Pml*^*−*/*−*^ cells. Immunostaining of CLOCK and PER2 revealed that the CLOCK distribution pattern was primarily cytosolic with a significant amount in a complex with PER2 (Fig. 2b). However, unlike the co-localization of PER2 and BMAL1, which appears as uniform overlapping foci, discrete foci of red (PER2) and green (CLOCK) were observed in the cytosol suggesting that CLOCK and PER2 may not directly interact (Fig. 2b). Co-IP studies followed by Western analysis indicated that cytosolic CLOCK IPs with PER2 in the presence of either K487R-PML or PML (Fig. 2f).

To examine these protein interactions further, we undertook three-color immunostaining for BMAL1, PER2 and CLOCK co-transfected into *Pml*^+/+^ or *Pml*^*−*/*−*^ MEF. The immunostaining pattern revealed that in wild type MEF, the majority of expressed PER2 was in the nucleus and was largely co-localized with CLOCK and BMAL1 (Fig. 2g). Interestingly, some of the cytosolic PER2 (red) appears independent of BMAL1 (blue) or CLOCK (green) raising the possibility that PER2 does not directly interact with BMAL1. In *Pml*^*−*/*−*^ MEF, nuclear CLOCK co-localized with BMAL1 indicating that BMAL1 can interact with CLOCK in the absence of PML. Western analysis of cell fractions indicated that PML has some influence on CLOCK but not BMAL1 nuclear localization. Together, these observations suggest that PML is not only important for PER2 nuclear localization but likely has a key role in recruiting PER2 to the BMAL1/CLOCK heterodimer complex.

### BMAL1 is pivotal for BMAL1/CLOCK heterodimer interaction with PML

**Figure 3:**
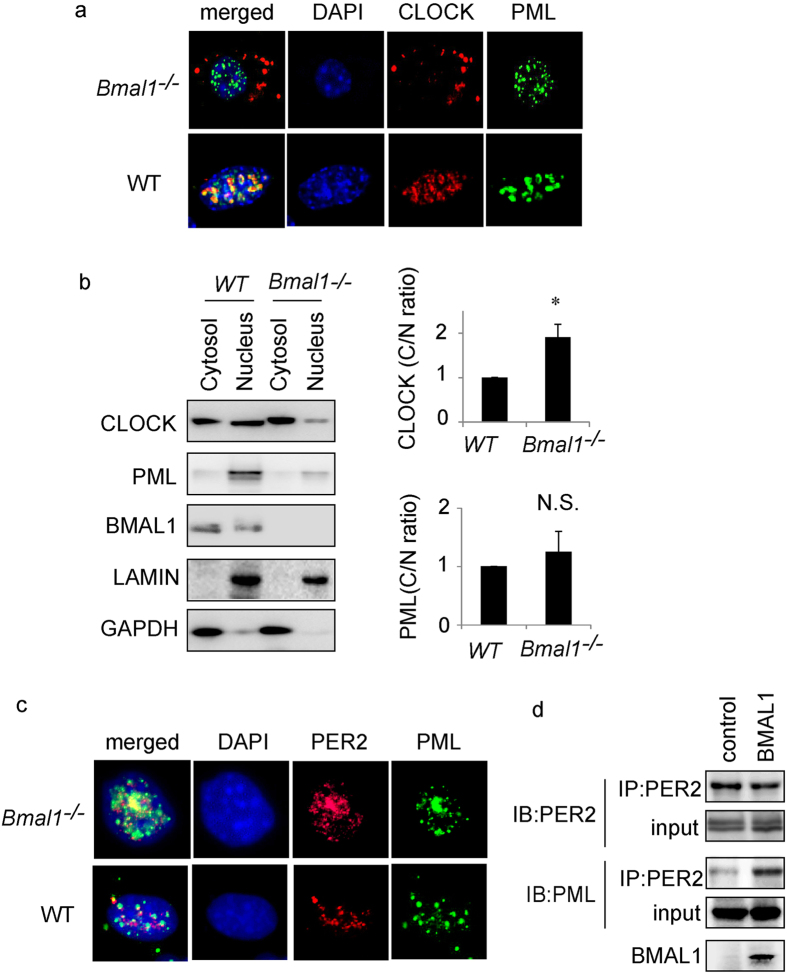
Immunofluorescence and immunoprecipitation analysis of BMAL1’s influence on PML and CLOCK nuclear localization and interaction. Immunofluorescence analysis of *Bmal1*^*−*/*−*^ and WT MEF cells transfected with plasmids expressing (**a**) Flag-CLOCK (red) and PML (green). We observed more than 20 cells and pictures are representative images. (**b**) Left: western analysis of cytosol and nuclear fractions for wild type and *Bmal1*^*−*/*−*^ cells using the respective antibodies for PML, CLOCK and BMAL1. Right: statistical analysis of cytosol (C)/nuclear fraction (N) ratio. WT MEF cells were set to 1.0. Bars: SEM. P < 0.05. N.S.: Not significant. n = 4. (**c**) Immunofluorescence analysis of *Bmal1*^*−*/*−*^ (upper) and WT MEF (lower) cells using the appropriate antibodies for PER2 and PML. We observed more than 20 cells and pictures are representative images. (**d**) Western analysis of IP from *Bmal1*^*−*/*−*^ cells transfected with the indicated plasmid and stained with the indicated antibodies as stated (n = 2).

The above IF distribution patterns suggest that an additional intermediary partner other than PML is necessary for CLOCK and PER2 to form a complex. We reasoned that this intermediary partner could be CLOCK’s natural heterodimer partner BMAL1. To investigate this possibility we transfected expression constructs for *FLAG-Clock* and *Pml* into *Bmal1*^*−*/*−*^ ear fibroblasts to examine whether PML co-localizes with CLOCK in the absence of BMAL1. IF staining with anti-FLAG and anti-PML antibodies revealed that the majority of FLAG-CLOCK staining in *Bmal1*^*−*/*−*^ cells was in the cytosol (Fig. 3a). In contrast, PML was detected primarily in the nucleus indicating that CLOCK and PML do not interact in *Bmal1*^*−*/*−*^ cells. To confirm the IF staining observations, we carried out cell fractionation followed by western analysis. The results revealed that CLOCK was largely cytosolic in *Bmal1*^*−*/*−*^ compared to *Bmal1*^+/+^ cells (Fig. 3b). In contrast, PML maintained its nuclear localization in BMAL1 deficient cells (Fig. 3a,b). These observations indicate that nuclear entry of CLOCK but not PML is regulated by BMAL1. These findings also indicate that CLOCK does not apparently co-localize or interact with PML in the absence of BMAL1.

Next, we carried out IF staining for native PER2 and PML in *Bmal1*^*−*/*−*^ cells. Immunostaining results show that both PER2 and PML were primarily localized in the nucleus (Fig. 3c). In addition, co-IP studies using *Bmal1*^*−*/*−*^ cells revealed that anti-PER2 antibodies pull down both PER2 (control) and PML (Fig. 3d). These findings suggest that PER2 and PML interact even in the absence of BMAL1.

## Discussion

Our current understanding of the mammalian molecular clock mechanism is based on the auto-feedback regulation of BMAL1/CLOCK mediated transcription. It is widely believed that PER2 regulates its own transcription via direct interaction with CRY1 to bind the BMAL1/CLOCK transcriptional heterodimer leading to repression of its promoter activity. Compelling evidence supporting this model has come from studies showing PER2 co-IPs with BMAL1 or CLOCK indicating that these proteins are in a complex. In addition, yeast two-hybrid assay studies also confirm that PER2 is likely in a complex with BMAL1/CLOCK. However, co-IP studies do not exclude the possibility that PER2 is interacting with BMAL1 and CLOCK via an intermediary partner(s) since co-IP only indicates that the proteins involved are in a complex. The yeast two-hybrid assay only requires activating and binding domains of proteins to be in close proximity for the assay to register a positive interaction outcome^13^. Hence the possibility that PER2 is in close proximity but not in direct contact with BMAL1/CLOCK is not excluded by these studies. Another feature of the auto-feedback mechanism is that CRY1 and CRY2 are essential for PER2 nuclear localization^14^. However, PER2 was observed in the nucleus of the SCN and peripheral tissues of CRY1 and CRY2 double deficient mice^15^. Furthermore, we observed a dominant cytosolic distribution of PER2 in *Pml*^*−*/*−*^ cells with normal expression of CRY1 and CRY2^8^. Thus, CRYs are not as essential for PER2 nuclear localization as previously suggested.

Our observations that PML regulates mammalian circadian behavior have led us to the molecular finding that PML is important for PER2 nuclear localization. We have observed that in the absence of PML, the localization of PER2, whether in the SCN or peripheral tissues, is largely cytosolic and is independent of temporal regulation^8^. This observation was further validated by co-IP studies showing that in the absence of PML, PER2 would not co-IP with either BMAL1 or CLOCK. These findings were initially surprising given that it is widely believed that PER2 directly interacts with BMAL1 and CLOCK. To investigate these observations further, we undertook the current studies using multicolor IF staining validated by co-IP and cell fractionation analysis to better understand the organization of PER2 interaction with BMAL1 and CLOCK. In the absence of PML, IF staining demonstrated that PER2 is heavily cytosolic while BMAL1 was biased towards the nucleus. Cell fractionation analysis revealed that BMAL1 localization is not dependent on PML since we observed a similar relative distribution of BMAL1 in the nucleus and cytosol from both *Pml*^+/+^ and *Pml*^*−*/*−*^ cells by Western analysis. By similar analysis, we observed that the cellular distribution of CLOCK is biased towards the cytosol over the nucleus in *Pml*^*−*/*−*^ cells, though to a lesser extent compared to that of PER2. Our studies suggest that PML influences the cellular distributions of these proteins to different degrees.

**Figure 4:**
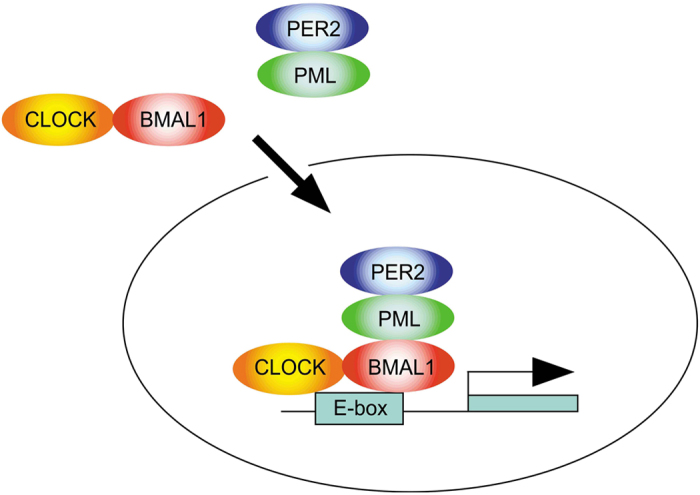
A model for the organization of PER2, PML, BMAL1 and CLOCK complexes.

To investigate whether PML could directly interact with CLOCK without BMAL1, we undertook co-localization studies in *Bmal1*^*−*/*−*^ cells, since our observations suggested that PML could directly interact with BMAL1. We observed that nuclear distribution of PML was not affected by the absence of BMAL1. However, CLOCK cellular distribution was primarily cytosolic in *Bmal1*^*−*/*−*^ cells indicating that BMAL1 regulates CLOCK nuclear localization. This observation is consistent with previous findings that BMAL1 is important for CLOCK nuclear entry^16^. This was confirmed by cell fractionation analysis showing that PML is primarily a nuclear protein whereas CLOCK was cytosolic in *Bmal1*^*−*/*−*^ cells. Based these observations, we conclude that PML and CLOCK likely do not directly interact. Our current observations are consistent with previous findings that BMAL1 co-localizes with PML suggesting that PML must directly interact with BMAL1^17^. Using a mutant K487R PML which is cytosol bound, we observed that CLOCK, BMAL1 and PER2 were co-localized in a complex in the cytosol. In the presence of wild type PML, CLOCK, BMAL1 and PER2 co-localized in the nucleus. In addition co-IP analysis, using anti-PER2 antibodies and extracts from *Pml*^*−*/*−*^ cells expressing K487R PML, demonstrated that mutant PML was equally efficient in pulling down BMAL1 as wild type PML. This suggests that K487R PML must directly interact with BMAL1. Since PML does not directly interact with CLOCK and PER2 does not directly interact with BMAL1 or CLOCK, we reasoned that organization of the complex requires PER2 to bind PML, PML to bind BMAL1 but not CLOCK and CLOCK to bind BMAL1 (Fig. 4). However, the presence of BMAL1 seems to enhance or stabilize PER2 and PML interaction (Fig. 3d). When all three proteins are present, their co-localization appears greatest (Fig. 2g) while PER2 and PML appear to only partially co-localize inside the nucleus when BMAL1 is missing (Fig. 3c).

The observations that PER2 does not directly interact with BMAL1 or CLOCK is in line with biochemical reconstitution studies with purified proteins showing that addition of PER2 unlike CRY1 did not alter the migration of the BMAL1/CLOCK:DNA complex in an electromobility shift (EMS) assay^7^. In these studies, it was observed that when CRY1 was associated with a BMAL1/CLOCK: DNA complex, the addition of PER2 altered the complex migration in the EMS assay suggesting that PER2 does not directly interact with BMAL1/CLOCK but requires an intermediary partner such as CRY1.

This study provides further evidence that PML is an important player in the circadian mechanism. To date, close to a hundred proteins in many diverse signaling pathways are known to interact with PML-NB’s, including p53, pRB, SIRT1, CBP, Rad51, BLM, WRN and SIN3A. Many of these proteins are known to be involved in the clock mechanism^18^,^19^,^20^,^21^. It is likely that PML-NB’s serve as macromolecular scaffolds that facilitate the interactions of components of the clock mechanism with regulators of various cellular functions.

In summary, our studies suggest that PML serves as an intermediary partner that recruits PER2 into the nucleus to bind the BMAL1/CLOCK complex. BMAL1 is the pivotal partner in the heterodimer transcriptional complex since it regulates CLOCK nuclear entry and interacts with PML thus allowing PER2 to interact with the complex.

## Materials and Methods

### Cell culture and plasmids

Wild type MEF and *Pml*^*−*/*−*^ MEF cells were established as described previously^8^. *Bmal1*^*−*/*−*^ ear fibroblast cells were established from adult *Bmal1*^*−*/*−*^ mice^22^ kindly provided by Dr. Loning Fu (Baylor College of Medicine, Houston). *Flag-Clock, HA-Bmal1, Per2, Flag-Per2*, *Pml* expression plasmids were described previously^8^. For the reconstitution of *hBmal1* in *Bmal1*−*/*−** cells, we prepared a Lentivirus *Bmal1* expression vector as follows. Full length h*Bmal*I was excised from the pCDNA-h*Bmal*I expression vector using *Swa*I and *Not*I and cloned into CSII-EF-IRES-Bsd, a kind gift from Dr. Hiroyuki Miyoshi at RIKEN, using *Swa*I-*Not*I sites^23^. Lentivirus vectors were prepared as described previously^23^. Lentivirus Infected *Bmal1*^*−*/*−*^ cells were then selected by Blasticidin for 10 days and cells were used after a few passages.

### Immunocytochemistry

Cells were seeded on glass bottom slides and the indicated plasmids were transfected using lipofectamine 2000 according to the manufacturer’s instructions. After 24 h, cells were treated with cytoskeleton buffer containing 10 mM piperazine‐N,N′‐bis(2‐ethanesulfonic acid (PIPES) pH 6.8, 100 mM NaCl, 300 mM sucrose, 3 mM MgCl_2_, 1 mM EGTA and 0.5% Triton X‐100 as described previously^8^. After washing with PBS, cells were fixed with 4% PFA and permeabilized with 1% Triton X-100/0.5% NP40/PBS. Primary antibodies were then applied for 2 h at room temperature (Rat anti-HA: Roche, Mouse anti-FLAG: Sigma, Mouse anti-mPML: Upstate, anti-hPML: Santa Cruz). After three washes with 1% triton X-100/0.5% NP40/PBS, secondary antibodies were applied (anti-Mouse 488 Alexa Fluor, anti-Rabbit 555 Alexa Fluor, Anti-rat 647 Alexa Fluor) for 1 h and observed with a fluorescence deconvolution microscope (Applied Precision, Issaquah, WA, USA).

### Immunoprecipitation and Immunoblotting

The cells were lysed with lysis buffer containing 50 mM Tris–HCl, pH 7.4, 0.1% SDS, 0.25 mM deoxycholate, 150 mM NaCl, 2 mM EGTA, 0.1 mM Na_3_VO_4_, 10 mM NaF, 1 mM PMSF, and complete Protease Inhibitor (Roche). The lysates were then sonicated and centrifuged to collect the supernatant. Immunoprecipitation was carried out using an anti-PER2 antibody overnight at 4 °C. Protein A (Pierce) was used to collect the antibody-protein complex and precipitates were washed three times with lysis buffer. Lysates and immunoprecipitates were separated by SDS-PAGE and Immunoblotting was performed as described previously^8^. We used anti-PER2^4^, anti-BMAL1 (abcam), anti-CLOCK (Thermo Scientific), anti-Lamin A/C (BD Bioscience), anti-GAPDH (Ambion), anti-HA (Cell signaling), anti-FLAG (Sigma), and anti-mPML (Upstate) antibodies.

### Cell fractionation

Cell fractionation was performed as described previously^24^. Cells were harvested with Buffer A containing 10 mM Hepes (pH 7.5), 1.5 mM MgCl_2_, 10 mM KCI and 0.5 mM dithiothreitol, homogenized and centrifuged to collect the supernatant as the cytoplasmic fraction. The precipitates were washed with buffer A three times and dissolved in Buffer C containing 20 mM HEPES (pH 7.5), 20% (vol/vol) glycerol, 0.42 M NaCl, 1.5 mM MgCl_2_, 0.2 mM EDTA, 0.5 mM phenylmethylsulfonylfluoride, and 0.5 mM dithiothreitol. Sonication and centrifugation were performed and the soluble fraction was collected as the nuclear fraction.

## Additional Information

**How to cite this article**: Miki, T. *et al*. Interactive Organization of the Circadian Core Regulators PER2, BMAL1, CLOCK and PML. *Sci. Rep.* **6**, 29174; doi: 10.1038/srep29174 (2016).
